# Chemistry and Biological Activities of Terpenoids from Copaiba (*Copaifera* spp.) Oleoresins

**DOI:** 10.3390/molecules17043866

**Published:** 2012-03-30

**Authors:** Lidiam Maia Leandro, Fabiano de Sousa Vargas, Paula Cristina Souza Barbosa, Jamilly Kelly Oliveira Neves, José Alexsandro da Silva, Valdir Florêncio da Veiga-Junior

**Affiliations:** 1 Chemistry Department, Amazonas Federal University, Av. Gal. Rodrigo Octávio, 6.200, Japiim, Manaus-AM, 69080-900, Brazil; 2 Graduate Program on Pharmaceutical Sciences, Paraíba State University, Rua Baraúnas, 351, Bairro Universitário, Campina Grande-PB, 58429-500, Brazil

**Keywords:** copaiba oil, oleoresin, sesquiterpenes, diterpenes, pharmacological activities

## Abstract

Copaiba oleoresins are exuded from the trunks of trees of the *Copaifera* species (Leguminosae-Caesalpinoideae). This oleoresin is a solution of diterpenoids, especially, mono- and di-acids, solubilized by sesquiterpene hydrocarbons. The sesquiterpenes and diterpenes (labdane, clerodane and kaurane skeletons) are different for each *Copaifera* species and have been linked to several reported biological activities, ranging from anti-tumoral to embriotoxic effects. This review presents all the substances already described in this oleoresin, together with structures and activities of its main terpenoids.

## 1. Introduction

The Copaiba oleoresin is obtained from the trunk of several *Copaifera *L. species (Leguminosae-Caesalpinoideae). These trees are native to the tropical regions of Latin America and Western Africa. There are more than twenty species occurring in the Brazilian territory, the most abundant being *C. officinalis *L., *C. guianensis *Desf., *C. reticulata *Ducke, *C. multijuga* Hayne, *C. confertiflora *Bth., *C. langsdorffii *Desf., *C.* *coriacea *Mart. and *C. cearensis *Huber ex Ducke [1,2,3].

Copaiba oleoresin is widely used as a popular medicine, through topical and oral administration. It has various ethnopharmacological indications, including: gonorrhea, bronchitis, pains in general, back pain, injury, blennorrhagia, leucorrhea, psoriasis, “catarro da bexiga”, wounds, asthma, as an antiseptic for wounds, skin ulcers, aching joints, ovarian cysts, uterine myoma, weak uterus, vaginal discharge, ovarian problem, ulcers, sore throat, uterine infections, general inflammations, as a tonic and to treat ulcers and other digestive diseases, and cancer, and leishmanioses [4,5,6,7,8,9,10,11,12].

Many studies have been performed in order to confirm these properties scientifically, and validate the widespread use of this oleoresin and its various pharmacological activities. Despite the many published papers, some of the data on the chemical composition and pharmacological activity of copaiba oleoresin remains contradictory. This study aims to expand knowledge about the chemical composition, biological activities and pharmacological actions of copaiba oleoresin and its major constituents.

## 2. Biological Studies with Crude Copaiba Oleoresins

Athough many species of *Copaifera* have been decribed, only nine of those have some biological study in the literature that evaluates the traditional uses. In some cases, these studies do not discriminate among the *Copaifera* species being studied, sometimes using commercial copaiba oleoresins. Table 1 shows all the biological and pharmacological activities that have been already tested for *Copaifera* oleoresins.

**Table 1:** Biological activities tested in different species of *Copaifera* oleoresins.

Species	Biological activity evaluated	Ref.
*C. cearensis* Huber ex Ducke	Antimicrobial	[13]
	Anti-inflammatory	[14]
	Antileishmanial	[15]
*C. duckei* Dwyer	antiproliferative	[16]
	Antimutagenic	[17]
	Embriotoxicity	[18]
	Anti-inflammatory	[19]
	Analgesic	[19]
*C. langsdorffii *Desf.	Antimicrobial	[13,20,21,22,23]
	Attenuation of ischemia/reperfusion-induced intestinal	[24]
	Gastroprotective effect on experimental gastric ulcer models in rats	[25]
*C. langsdorffii *Desf.	Ischemia-Reperfusion of Randomized Skin Flaps	[26]
	Antileishmanial	[15]
	Wound Healing	[27,28,29]
	Antioxidant	[30]
	Insecticide	[31]
	Anti-inflammatory	[32,33]
	Antimutagenic	[34]
*C. lucens* Dwyer	Antimicrobial	[13]
	Antileishmanial	[15]
*C. martii* Hayne	Antimicrobial	[13]
	Antileishmanial	[15,35]
*C. multijuga* Hayne	Anti-inflammatory	[14,36,37,38]
	Antimicrobial	[13,39,40,41,42]
	Antitumor	[43,44]
	Antinociceptive	[36,45]
	Antileishmanial	[15]
	Healing	[46]
*C. officinalis* (Jacq.) L.	Antimicrobial	[13,22,23,47,48]
	Antischemic	[49]
	Anti-inflammatory	[50]
	Antileishmanial	[15]
	Inhibition of human leukocyte elastase	[51]
	Effect Antitumor (Walker 256 carcinoma)	[52]
*C. paupera* (Herzog) Dwyer	Antimicrobial	[13]
	Antileishmanial	[15,53]
*C. reticulata* Ducke	Antiinflammatory	[14]
	Antimicrobial	[13,54]
	Inseticide	[55,56,57,58]
	Antinociceptive	[45]
	Teratogenicity and embriotoxicity	[59]
	Toxicity	[60]
	Antileishmanial	[15,61]
	Wound Healing	[62,63]
	Anxiolytic	[64]
*C. *sp. (commercial copaiba oleoresins)	Antimicrobial	[40]
	Anti-inflammatory	[65,66]
	Skin perfusion	[67]
	Insecticide	[68,69,70,71]

Of the various ethnopharmacological indications of copaiba oleoresins, some, such as anti-inflammatory, wound healing, antimicrobial, antileishmanial, larvicidal, antineoplasic and antinoceptive activities have been confirmed by pharmacological studies, as will be detailed below.

The very first study aiming to demonstrate the anti-inflammatory activity of copaiba oleoresin was performed by Basile *et al*. [65]. The activity found was related to the anti-oedematogenic effect observed in carragenin induced rat paw oedema, and was later confirmed by Veiga, Jr. *et al*. [66]. This later study also showed that the activity varies with copaiba oleoresins from different species, and using different flogistic agents. All these studies were performed with commercial copaiba oleoresins, but without identifying the individual species. Baylac and Racine [50] showed that *C. officinalis* oleoresin causes *in vitro *inhibition of 5-lipoxygenases, an important enzyme of the inflammatory cascade. The same authors [51] showed that the copaiba oleoresin obtained from *C. officinalis* was not capable of causing *in vitro* inhibition of HLE (Human leukocyte elastase), one of the main proteases in the neutrophils, which play an important role in the pathogenesis of many inflammatory disorders.

Veiga, Jr. *et al.* [14] evaluated the anti-inflammatory activity of three different copaiba oleoresins (*C. multijuga *Hayne, *C. cearensis *Huber ex Ducke and *C. reticulata* Ducke), and demonstrated that although similar in composition, they showed different activities. The assay was evaluated *in vitro* by measuring NO production by murine macrophages and *in vivo* using the zymosan induced pleurisy model in mice. The *C. multijuga *Hayne oleoresin was the most potent, inhibiting the NO production at a low concentration (5 µg/mL). The oleoresins from *C. cearensis *and *C.* *reticulata *presented similar activities but with less intensity (50 µg/mL and 500 µg/mL, respectively). Veiga, Jr. *et al.* [37] evaluated and afirmed that the crude *C. multijuga* Hayne oleoresin and its fractions (hexane, dichloromethane and methanolic) have anti-inflammatory properties against carrageenan- and bradykinin-induced oedema formation in the rat paw. Gomes *et al*. [36] suggest that *C. multijuga* Hayne oleoresin has anti-inflammatory activity by inhibiting histamine and the serotonine pathways. The *C. duckei* Dwyer oleoresin demonstrated anti-oedematogenic effect observed on carragenin induced rat paw oedema [19]. Araujo, Jr. *et al.* [49] studied the anti-inflammatory activity of *C. oficinalis* oleoresin in aminotransferases in rats submitted to hepatic ischemic and reperfusion, and found that it presented no activity. In a similar study, Brito *et al*. [38] evaluated the effect of the oleoresin of *C. multijuga* on urea and creatinine serum levels in rats submitted to ischemia and reperfusion kidney. They observed a decrease in vascular permeability to proinflammatory agents caused by copaiba oleoresin, when in turn, decreased the migration of toxic agents to the renal parenchyma, thereby mitigating the damage of this organ. Nogueira Neto *et al*. [33] tested the *C. langsdorffii* oleoresin on endometriosis foci in female rats, and found significant histological changes, with a reduction in volume of the endometrioses. 

The strong healing activity of copaiba oleoresins is one of the properties most frequently cited in ethnopharmacological studies. Despite this fact, the pharmacological studies are controversial. Brito *et al*. [62,63] observed that wounded rats treated with copaiba oleoresin obtained from *C. reticulata* took longer to heal and showed more inflammation than the control animals (saline). Similar results were obtained by Vieira *et al*. [28], who found that copaiba oleoresin from *C. langsdorffii *impairs the normal process of wound repair in the presence of a foreign body. Westphal *et al*. [46] observed an increase in tissue inflammation in rats treated by intrapleural injection of *C. multijuga* oleoresin. Also, according to Comelli, Jr *et al*. [29] the crude *C. langsdoffii* oleoresin has no effect on wound healing in intestinal mucosa of rats oil-treated orally. On the other hand, Paiva *et al.* [27] investigated the activity of wound healing in rats treated with copaiba oleoresin from *C. langsdorffii*, and obtained results that allowed them to affirm the benefits of this copaiba oleoresin, justifying its traditional use. These contradictory results may be due to the fact that oleoresins have different sources, because depending on environmental factors, plants produce different metabolites that can directly influence the activity [72].

Antimicrobial activity of oleoresin of copaiba is one of the most frequently studied properties, and various works have evaluated its antimicrobial activity against the following bacteria: *Escherichia coli* [21,39], *Staphylococcus aureus* [13,20,23,39,40,54], *Pseudomonas aeruginosa *[23,39], methicillin-resistant *S. aureus *[13], *Listeria monocytogenes* [21], *Staphylococcus epidermidis *[13], *Bacillus subtilis *[13,20,40], *Streptococcus mutans* [47], *Streptococcus salivarius *[47], *Streptococcus pyogenes *[47], *Proteus mirabilis* [13,23], *Klebsiella pneumoniae *[13,23], *Shigella flexinerii* [13,23], *Enterobacter cloacae* [13], *Enterococcus faecalis *[13,47], *Citrobacter freundi* [23], *Actinobacillus pleuropneumoniae* [23], *Haemophilus parasuis* [23], *Paenibacillus alginolyticus* [48], *P. pabuli* [48], *P. azotofixans* [48], *P. borealis* [48], *P. gluconolyticus* [48], *P. validus* [48], *P. thiaminolyticus* [48] and *P. larvae* [48]; yeasts: *Candida albicans* [13], *C. parapsilosis* [13,41,42], *C. tropicalis* [13,41,42] and *C.* *guilliermondii *[41,42]; and fungi: *Aspergillus flavus* [41,42], *A. niger *[41,42], *A. tamari *[41,42], *A. terreus *[41,42], *Trichophyton rubrum *[13], *T. mentagrophytes *[13], *Microsporum canis* [13] and *M. gypseum* [13].

Copaiba oleoresin from *C. multijuga* showed antimicrobial activity against *E. coli*, *S. aureus* and *P. aeruginosa* [39]. However, Pacheco *et al*. [40] did not observe any activity of the copaiba oleoresin from *C. multijuga* against *S. aureus* (or against any other bacteria analyzed). The authors also mention that another copaiba oleoresin (species not identified) showed no activity against *B. subtilis* and *S. aureus*. The fungicidal activity fungicida of *C. multijuga *oleoresin *in natura*, and a volatile fraction obtained of the hydrodistillation of this oleoresin, were evaluated *in vitro* against filamentous fungi (*Aspergillus*) and yeast (*Candida*). Samples were compared with the antibiotic drug Miconazole nitrate (MIC = 0.1–0.5 µg/mL), the volatile fraction being more active (MIC = 0.08–0.5 µg/mL) [41,42]. 

Oleoresins obtained from the species *C. martii*, *C. officinalis* and *C. reticulata* showed *in vitro* bactericidal activity against *S. aureus*, methicillin-resistant *S. aureus*, *S. epidermidis*, *B. subtilis*, and *E. faecalis *with minimum inhibitory concentrations ranging from 31.3–62.5 μg/mL [13]. The oleoresin from *C. reticulata* showed high activity against *S. aureus *multidrug resistant (MIC = 2.5 µg/mL) and *S. aureus *ATCC strains (MIC = 5.0 µg/mL) [54]. Pieri *et al.* [47] showed the ability of the *C. officinalis* oleoresin to inhibit bacterial adhesion in dog’s teeth by clinical and microbiological trials. 

Antimicrobial activity of two solutions containing oleoresins of two different species of *Copaifera* was tested against 27 strains of *Escherichia coli* obtained from mastitic milk of animal origin. The solution of *C. langsdorffii* oleoresin inhibited the growth of eight strains and another solution containing *C. officinalis* inhibited the growth of seven isolates. The results of this study suggest that the copaiba oleoresin may be a potential source of new and selective antimicrobial agents [22]. Pieri *et al*. [21] found that *C. langsdorffii *oleoresin did not alter its antimicrobial activity against bacteria of the *Listeria monocytogenes* species after exposure to high temperatures in an autoclave. In another study, two copaiba oleoresins were evaluated for antibacterial activity against pathogenic species of interest to animal and human health. The *C. langsdorffii* and *C. officinalis* oleoresins showed activities against *E. coli*, *P. aeruginosa*, *S. flexneri* and *S. aureus* [23]. Santos *et al*. [48] demonstrated that the essential oil of *C. officinalis* presents high activity against *Paenibacillus* species. These results show that some copaiba oleoresins have antimicrobial activity, confirming the findings of the ethnopharmacological studies. 

The studies of Paiva *et al. *[25] with *C. langsdorffii* copaiba oleoresin found a reduction in gastric wounds induced by ethanol, and a hypothermic restraint-stress in the indomethacin model mediated through its effect on mucus production and by its antiacid secretory properties. Later, Paiva *et al.* [24] demonstrated the protector activity of this oleoresin against ischemia/reperfusion-induced intestinal tissue damage.

The anticancer activity of copaiba oleoresins from some species has been studied using diverse models. The *C. multijuga *oleoresin and its (hexane and chloroform) fractions obtained by fractionation using KOH impregnated gel column chromatography demonstrated significant inhibitory effect on Erlich tumor-bearing mice [44], and found that it reduced the growth of melanoma cells on mice [43], both after oral administration, confirming its use by traditional medicine. However, Brito *et al.* [52] found that the species *C. officinalis* stimulated the tumor growth of Walker 256 carcinoma inoculated into the vagina and uterine cervix of rats. 

Gomes *et al.* [45] observed a central and peripheral antinociceptive activity in two copaiba oleoresins (*C. multijuga* and *C. reticulata*), and suggest that fractions (hexane, chloroform and methanol) obtained from *C.* *multijuga* oleoresin after a KOH impregnated gel column chromatography have antinociceptive effect mediated by the opioid receptors [36]. Carvalho *et al.* [19] demonstrated the existence of analgesic activity from *C. duckei* Dwyer oleoresin by intraperitoneal administration of acetic acid solution in mice.

The mutagenic and cytotoxic activity of *C. langsdorffii* oleoresin were evaluated in erythrocytes of *Mus musculos* mice treated with crude oleoresin by oral administration, in which dose-dependant toxic capacity was found [34]. The mutagenic and cytotoxic activities of *C. duckei* oleoresin were evaluated in Wistar rats by dermal application, and was found to have no toxicity to the peripheral blood reticulocytes and bone marrow cells [17]. In another study, the acude toxic and neurotoxic effects of *C. reticulata* oleoresin administered orally to the Wistar rats species were evaluated, presenting low mortality and a very high toxic dose [60]. The same author evaluated the embriotoxicity of oleoresin from *C. reticulata* in pregnant rats. The oleoresin was toxic to the mother and embryotoxic, but not letal at any dose level [59].

Lima *et al*. [18] performed a pre-clinical trial in Wistar rats (*Rattus norvegicus*) of a vaginal cream containing 2.5% of *C. duckei* oleoresin. This study demonstrated the absence of maternal toxicity, embryofoetotoxicity and fetotoxicity at the dose administered (10 times that recommended in humans), and it was concluded that the vaginal cream is safe during pregnancy.

The anxiolytic activity was evaluated in an ethological study in rats treated with *C. reticulata* oleoresin. The studies demonstrated that copaiba oleoresin produced dose-dependent anxiolytic-like effects across all the dose ranges tested, within conventional and ethological parameters, without adversely affecting general activity [64].

Many studies still report insecticidal activity of great interest in popular knowledge, such as *C. reticulata* oleoresin tested for its insecticidal activities against the Japanese termite (*Reticulitermes speratus* Kolbe) using a fumigation bioassay, which did not demonstrate insecticidal activity [57]. 

A study evaluated the use of commercially available insect repellents used by military personnel in a jungle environment in the Amazon region. The repellent DEET (*N,N*-diethyl-3-methylbenzamide) was compared with natural oil-repellents containing *Copaifera spp.* oleoresin. The results showed a higher degree of perceived protection against damage caused by insects with the repellant containing copaiba oleoresin [68]. 

The acaricidal activity of oleoresinous extract from *C. reticulata* was investigated; larval mortality was tested after treatment with a solution containing the oleoresin, and the concentration was evaluated to determine lethal concentrations [58]. 

Larvicidal activity of *C. reticulata *oleoresin was observed for *Culex quinquefasciatus*, the main transmitter of *Bancroftian filariasis *[56]. Copaiba oleoresins from *C. langsdorffii* showed significant activities against *Aedes aegypti* (LC_50_ = 41 µg/L) in the larvicidal assay [31]. Another study demonstrated the potential of *Copaifera spp.* oleoresin to inhibit *A. aegypti* proliferation, showing larvicidal activity at low concentrations (LC_50_ = 44–51 mg/mL), and a gradual reduction in activity was observed over several days [69]. Silva *et al*. [55] showed that the hexanic and methanolic fractions of oleoresin from *C. reticulata* exhibited high toxicity against *A. aegypti* larvae. Prophiro *et al*. [71] investigated the efficiency of solution prepared with *Copaifera spp.* oleoresin as a larvicide in wild populations of *A. aegypti* with resistence to organophosphate, showing larvicidal activity in all concentrations tested. The same author studied the start time of larvicidal activity, residual effect, and the effect of very low concentrations of this oleoresin on *A. aegypti*; the results demonstrated a lethal effect between the first 2 and 3 h of larval development, with the toxic effect remaining totally effective (100% mortality) until the sixth day for *Copaifera sp.* (90 mg/L) [70].

Santos *et al.* [15] screened eight different Brazilian copaiba oleoresins for antileishmanial activity, and observed a variable level of activity against *Leishmania amazonensis* (IC_50_ = 5.0 to 22 µg/mL), with the oleoresin from *C. reticulata *showing the strongest antileishmanial activity (IC_50_ = 5 μg mL^−1^) for promastigote forms of *L. amazonensis* after 72 h of incubation. Kvist *et al*. [53] observed moderate leismanicide activity for *C. paupera* oleoresin (IC_50_ = 17 µg/mL), lower than that found by Santos *et al.* [15], that was IC_50_ = 11 µg/mL. The oral treatment with *C. martii* oleoresin showed a significant reduction in the average lesion size (1.1 ± 0.4 mm) caused by *L. amazonensis* when compared with untreated mice (4.4 ± 1.3 mm), and histopathological evaluation did not reveal any changes in the animals treated with copaiba oleoresin, compared with the control animals. In this study, morphological and ultrastructural analyses demonstrated notable changes in parasite cells treated with this oleoresin [35]. Significant antileishmanial activity of copaiba oleoresin from *C. reticulata* was demonstrated against axenic amastigote (IC_50_ = 15.0 μg/mL) and intracellular amastigote (IC_50_ = 20.0 μg/mL), forms of the parasite *L. amazonensis* [61].

Copaiba oleoresin is also used by the cosmetics and varnish industries [12]. Oliveira *et al.* [67] observed that copaiba oleoresin has potential for use in topical formulation, as a stimulant agent for the absorption of hydrofilic bioactive substances. Despite the high volume of published works on copaiba oleoresin and its biological activities, there are few references that identifiy the compounds responsible for its biological activity. 

## 3. Chemical Composition of Copaiba Oleoresins

Copaiba oleoresin is a transparent liquid with variable colour and viscosity. It consists of a mixture of sesquiterpenes and diterpenes. The oldest chemical study with copaiba oleoresin dates back to the beginning of the 19th century, when Schweitzer, in 1829, described how copaiba oleoresin, when left standing, turned into a solid substance and crystallized. He called this substance copaivic acid [12]. It is difficult to say precisely what the structure of this substance was, due to the lack of information described, and the unavailability of identification techniques at that time. 

A review article from 2002 listed the sesquiterpenes and diterpenes described in the literature on copaiba oleoresins [12]. New substances and other undescribed terpenoids have been published since then. At least 38 other sesquiterpenes were identified. Of these, 35 were found in oleoresins of *C.* *duckei*, *C. paupera*, *C. piresii*, *C. pubiflora *and *C. reticulata*: cyclosativene [73,74,75,76], 7-episesquithujene [76], cyperene [76], *cis*-α-bergamotene [73,76], *trans*-α-bergamotene [73,74,75,76], (Z)-β-farnesene [75,76], guaia-6,9-diene [76], *epi*-β-santalene [73,76], (*E*)-β-farnesene [73,76], sesquisabinene [76], 4,5-diepiaristolochene [76], germacrene A, [74], *trans*-cadina-1(6),4-diene [74], β-chamigrene [73,76], *cis*-β-guaiene [76]; viridiflorene [76], γ-gurjunene [73], γ-curcumene [73], *epi*-cubebol [74], valencene [73,76], trans-β-guaiene [76], (*E,E*)-α-farnesene [76], (*Z*)-α-bisabolene [73,75,76], α-bulnesene [73], β-curcumene [73], (*Z*)-γ-bisabolene [76], 7-*epi*-α-selinene, [74,75,76], *trans*-cadina-1(2),4-diene [74], (*E*)-γ-bisabolene [73,75,76], globulol [74], humulene epoxide II [75], *epi*-cubenol [74], cubenol, [74], *epi*-α-muurolol [74] and *epi*-β-bisabolol [73]. From hydrodistillation of the *C. langsdorffi* and *C. martii* oleoresins, a further identified three sesquiterpenes were identified: seline-3,7(11)-diene [77], α-calacorene [78] and gleenol [78]. Even with the great variation that the chemical composition of these oleoresins usually presents, β-caryophyllene, considered a chemical marker of these oleoresins, is usually the major constituent [79]. However, α-copaene was the major constituent of samples of *C. paupera* and *C. piresii* oleoresins collected in Acre and Rondônia, respectively [74], and was also the major constituent in the samples of *C. martii* oleoresins collected in Pará, subjected to hydrodistillation [78]. Meanwhile, β-bisabolene was the major constituent in several samples of *C. duckei *and *C. reticulata* collected in Pará [73,75].

As for the diterpenes, at least 15 other diterpenes not reported in the review article of 2002 were identified, including four with kaurane-type skeletons: *ent*-kaur-16-ene [80], *ent*-kaur-16-en-19-al [80], 19-*nor*-kaur-16-en-4α-ol [80,81] and *ent*-kaur-16-en-19-ol [80]; three of clerodane-type skeleton: clerodan-15,18-dioic acid [82], 7α-acetoxyhardwickiic acid [83] and 7α-acetoxybacchotricuneatin D [84]; and eight with labdane-type skeletons: *ent-*4-epi-agathic acid [81], 3-hydroxycopalic acid [85], 3-acetoxy-copalic acid [79], 14, 15-dinorlabd-8(17)-en-13-one [86], (-)-3-β-hydroxy-15,16-dinorlabd-8(17)-ene-13-one [84], (-)-15,16-dinorlabd-8(17)-en-3β,13-diol [87], (-)-13(*R*)-14,15-dinorlabd-8(17)-ene-3,13-diol [88] and pauperol [86].

There are some doubts as to the exact structures of these two last diterpenes. The *ent*-dinorlabdane (-)-13(*R*)-14,15-dinorlabd-8(17)-ene-3,13-diol, was isolated from a commercial copaiba oleoresin [88]. This substance may have been degraded from 3-hydroxycopalic acid, since the oleoresin, without identification, would have been exposed to light and temperature conditions that could lead to loss of part of the side chain. The other is a C35 methyl ester, a substance produced by the coupling of a labdanoic diterpene and a sesquiterpene alcohol, giving the ester pauperol, isolated from *C. paupera *[86]. Indeed, this substance may not be originally present in the oleoresin, since the authors report that they performed esterification (diazomethane) prior to the isolation.

The perfume and cosmetics industries have shown great interest in the sesquiterpene fraction, which is responsible for the aroma of copaiba oleoresin. The commercial value of concentrates of sesquiterpenes of *Copaifera *is as much as six hundred times higher than that of the whole copaiba oleoresin. Sant’Anna *et al*. [89] evaluated the volatile fraction of *C. multijuga* oleoresin and indicated minor compounds such as δ-cadinene, δ-cadinol, (*Z*)-α-santalol, caryophyllene oxide, α-cadinol and τ-muurolol as the most intense compounds in the aroma of the copaiba oleoresin studied. Chiral GC-O-MS proved (+)-δ cadinene to be the only enantiomer present in the oleoresin, with a sweet, green, refreshing aroma.

### 3.1. Pharmacological Activities of the Main Sesquiterpenes from Copaiba Oleoresin

Many studies have shown that sesquiterpenes are the main substances present in copaiba oleoresins. Sometimes, these account for more than 90% of their composition. Because they are the major components, many of the pharmacological activities of copaiba oleoresins are attributed to the main sesquiterpenes from the oleoresin. However, the pharmacological effect of the oleoresin, cannot be attributed to just one constituent, because the constituents present in oleoresin may interact synergistically in the promotion of the activity observed.

**Figure 1:**
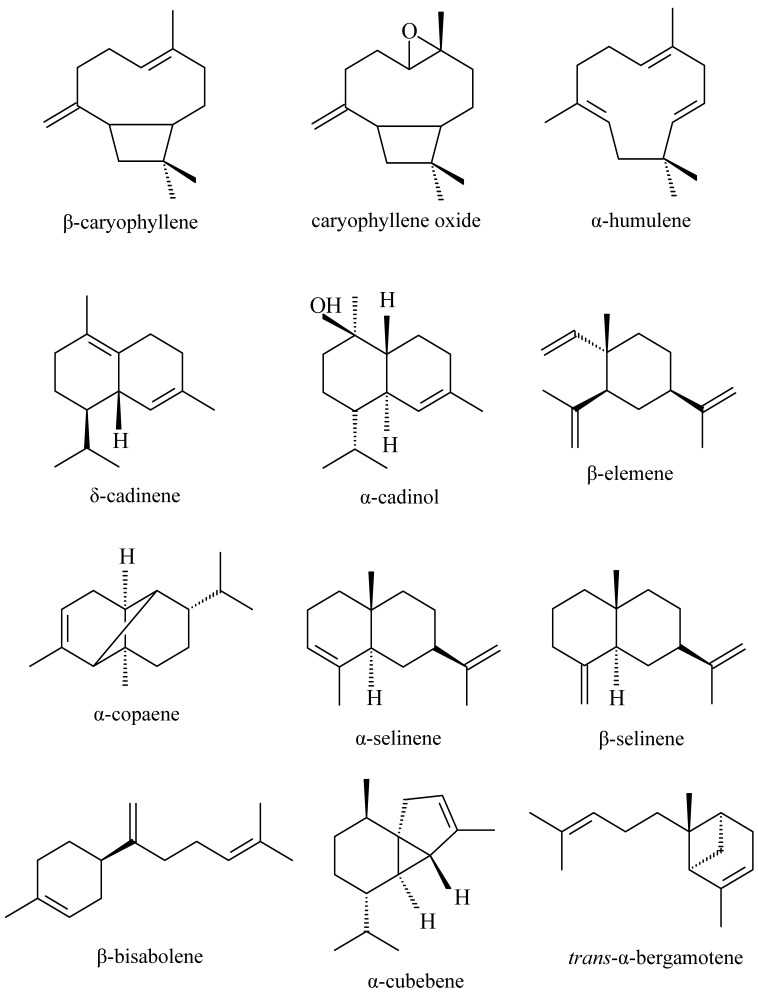
Main sesquiterpenes detected in copaiba oleoresin.

The main sesquiterpenes found in copaiba oleoresins are: β-caryophyllene, caryophyllene oxide, α-humulene, δ-cadinene, α-cadinol, α-cubebene, α- and β-selinene, β-elemene, α-copaene, *trans*-α-bergamotene, and β-bisabolene (Figure 1).

Some of the sesquiterpenes are major components in the oleoresin and others, although present in minor proportions, are often detected. Some studies of the pharmacological activities of some of the main sesquiterpenes found in the copaiba oleoresin are described below. 

The sesquiterpenes β-caryophyllene and its oxide are both commonly found in copaiba oleoresins and in many other plant species [90,91]. Cascon *et al*. [79] suggested that caryophyllene oxide is possibly an oxidative artefact produced during storage of oleoresin. Several biological activities are attributed to β-caryophyllene, such as insecticidal [92,93], antimicrobial [94,95], local anaesthetic [96], anticarcinogenic [97,98,99,100,101], and anti-inflammatory [90,91] activities.

Rodilla *et al*. [92] isolated β-caryophyllene and its oxide from *Laurus novocanariensis* leaves essential oil and they showed that both were strong antifeedants to *Leptinotarsa decemlineata* and *Spodoptera littoralis*. Another study demonstrated the repellent effect of caryophyllene oxide against *Anopheles* *gambiae* [93]. The sesquiterpenes β-caryophyllene and caryophyllene oxide, isolated from the oil of *Calocedrus formosana *leaves, presented antitermitic activity and antifungal activity against *L. sulphureus *[95]. Goren *et al*. [94] reported that β-caryophyllene presented antimicrobial activity against *E. coli*, *S. aureus*, *K. pneumonia*, *P. aeruginosa* and *C. albicans*, and caryophyllene oxide showed activity only for *C. albicans*. Ghelardini *et al*. [96] demonstrated that β-caryophyllene has a strong local anaesthetic action.

Many authors have reported the anticarcinogenic properties of β-caryophyllene. According to Silva *et al.* [97] and Kubo *et al*. [98], this sesquiterpene exhibits cytotoxic activity against several solid tumor cell lines. A previous study showed that β-caryophyllene exhibited antiproliferative activity in human renal adenocarcinoma and amelanotic melanoma cells [102]. Futhermore, β-caryophyllene has also been reported to increase the anticancer activity of α-humulene, isocaryophyllene and paclitaxel against tumour cell lines [101]. In study by Zheng *et al*. [99], the compounds: β-caryophyllene, β-caryophyllene oxide, and α-humulene (all present in the copaiba oleoresins) showed significant activity as inducers of the detoxifying enzyme glutathione S-transferase in mouse liver and small intestine. Finally, antimutagenic activity of the β-caryophyllene was observed by Di Sotto *et al*. [100]. According to the authors, this sesquiterpene was able to protect human lymphocytes cultivated with genotoxic damage induced by ethylic methanesulfonate and colcemid.

Many studies have also confirmed the anti-inflammatory activity of β-caryophyllene and/or caryophyllene oxide. Tung *et al*. [90] studied anti-inflammatory activities of essential oil from *Cinnamomum* *osmophloeum* twigs and its main constituents. In this study, the sesquiterpenes β-caryophyllene and its oxide exhibited excellent anti-inflammatory activities in suppressing nitric oxide production by LPS-stimulated macrophages. In other studies, caryophyllene oxide showed significant central as well as peripheral analgesic, along with anti-inflammatory, activity [91] and showed inhibitory effect on histamine-induced contraction in guinea pig ileum [103]. Cho *et al*. [104] demonstrated the ameliorative effect of oral administration of β-caryophyllene in mice on experimental colitis induced by dextran sulfate sodium. 

The sesquiterpenes β-caryophyllene and α-humulene, isolated from *Cordia verbenacea* leaves essential oil, showed systemic anti-inflammatory activity in rat paw oedema induced by carregeenin, bradykinin, P Substance, histamin and plaquetary activating fator (PAF), and also oedema induced by *Apis mellifera* venom or ovalbumin in sensitized rats. In the same paper, a decreasing in TNF level was observed, without this affecting the production of interleukin-1 [105]. The link between isolated α*-*humulene and β-caryophyllene and the release of inflammatory mediators, such as bradykinin, FAP, histamine, interleukin-1β, TNF and prostaglandins, was observed, together with COX-2 inhibition NF-KB [106]. 

Likewise, a solution of α-humulene and β-caryophyllene showed action against allergy-related inflammation in an experimental model in which this solution was used to treat mice sensitized with oral or nasal administration. α-Humulene showed activity even in therapeutic or preventive treatments, reducing the eotaxin and interleukin-5 levels of the mediastine lymph nodes (*in vitro*), a result not shown for β-caryophyllene. α-Humulene also reduced the nuclear transcription factor (NF-KB), P-selectin expression in the lung tissue and mucus secretion from the lungs, results that suggest its potential use for the treatment of asthma and allergy-related inflammatory diseases [107]. Furthermore, α-humulene showed cytotoxity activity against several solid tumor cell lines, including breast cancer adenocarcinoma, prostatic adenocarcinoma, lung carcinoma, colon adenocarcinoma lines, and human melanoma cell line, besides mouse colon cell line. The authors suggested that the cytotoxicity of α-humulene resulted in cellular glutathione depletion and reactive oxygen species production [108]. 

Other common sesquiterpenes in the copaiba oleoresin are δ-cadinene and α-cadinol. δ-Cadinene inhibited the growth of *Streptococcus mutans* (one of the most important cariogenic bacteria) and *Propionibacterium acnes* (one of the bacteria responsible for acne) [109]. Pérez-Lopez *et al.* [110] performed a bioassay-guided fractionation of the essential oil obtained from the fruit of *Schinus molle* against *S. pneumonia* resistant to conventional antibiotics, which led to the identification of δ-cadinene as the principal active constituent (MIC of 31.25 μg/mL) from the oil. 

Previous studies have reported that α-cadinol showed antitermitic activity [95], insecticidal activity against yellow fever mosquito larvae, and was selectively cytotoxic against human colon adeno-carcinoma [111]. Furthermore, it exhibited antifungal activity against *C. versicolor *[112] and *L. sulphureus *[95,112].

Elemene is mainly composed of β- and δ- and γ-elemene, with β-elemene accounting for 60%–72% of all three isoforms [113]. β-Elemene is a broad-spectrum antitumor agent. It has been shown that this sesquiterpene is an effective treatment for various types of cancer, including gastric [114], lung [115], laryngeal [116], ovarian [117], brain [118], prostate [119] cancer, and leukemia [120].

Liu *et al*. [114] investigated the anti-tumor effect of β-elemene on human gastric cancer cells, and the molecular mechanism involved. The data provides the first evidence that β-elemene induces protective autophagy and prevents human gastric cancer cells from undergoing apoptosis. Wang *et al*. [115] indicated that human lung carcinoma cells were more sensitive to β-elemene than the others. 

The inhibitory effects and mechanism of elemene were also investigated in the growth of laryngeal cancer cells *in vitro* and *in vivo*, transplanting cell subcutaneously to BALB/c nude mice to produce solid tumors. Increased apoptosis was observed in elemene administered cells. *In vivo*, the growth of HEp-2 cell-transplanted tumors in nude mice was inhibited by intraperitoneal injection of elemene [116].

Li *et al*. [117] showed that β-elemene inhibited the proliferation of cisplatin resistant human ovarian cancer cells and their parental cells, but had only a marginal effect in human ovary cells, indicating differential inhibitory effects on cell growth when comparing ovarian cancer cells with normal ovary cells. It was also demonstrated for the first time that β-elemene markedly enhanced cisplatin induced growth inhibition in resistant cells compared to sensitive cells.

β-Elemene induced the formation of apoptotic bodies and DNA ladder on K562 leukemia cells, an effect that was dose- and time-dependent in β-elemene treated cells as compared with the untreated control cells [120]. Moreover, β-elemene has been shown to antagonize glioblastoma (the most prevalent type of primary brain tumor in adults) cells by inducing apoptosis disrupting the formation of a key step in maintaining the conformation stability of Hsp90/Raf-1 complex [118].

Another study was performed to assess the effect of β-elemene on prostate cancer cells, as well as other types of tumour cells, and to determine whether the effect of β-elemene on prostate cancer cell death was mediated through the induction of apoptosis. It was demonstrated that β-elemene suppresses the growth and proliferation of prostate cancer cells and other types of tumour cells *in vitro *[119].

Inhibition of cell proliferation [121] and induction of apoptosis [122] have been proposed as the underlying mechanism of the anti-tumor effects of β-elemene. Furthermore, several studies have indicated that β-elemene enhances the cytotoxic effect of radiation *in vitro* and *in vivo*. Li *et al*. [123] suggested that β-elemene can enhance lung (A549) cell radiosensitivity through the enhancement of DNA damage and the suppression of DNA repair.

### 3.2. Pharmacological Activities of the Main Diterpenes from Copaiba Oleoresin

**Figure 2:**
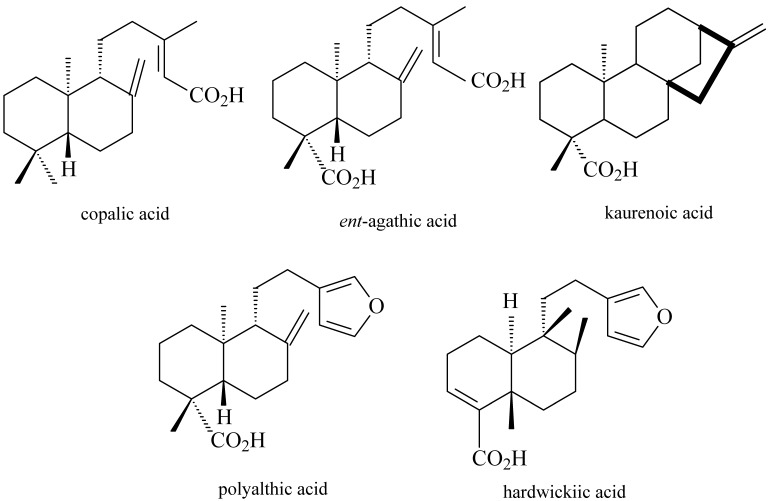
Main diterpenes detected in copaiba oleoresin.

The diterpenes most commonly found in copaiba oleoresins are copalic, polyalthic, hardwickiic, kaurenoic and *ent*-kaurenoic acids, together with their derivatives 3-hydroxy-copalic, 3-acetoxy-copalic, and *ent*-agathic (Figure 2).

Copalic acid was first described by Nakano and Djerassi [124], who isolated it from *Hymenea courbaril *resin samples. This diterpene is considered a biomarker for this genus *Copaifera* and some studies have been performed to evaluate the antibacterial activities of this substance. It has been demonstrated to have significant antimicrobial activity against *B. subtilis*, *S. aureus*, and *S. epidermidis* [86]. Recently, Souza *et al.* [125] investigated the antimicrobial activity of four labdane-type diterpenes [(-)-copalic acid, (-)-acetoxycopalic acid, (-)-hydroxycopalic acid and (-)-agathic acid] isolated from the copaiba oleoresin from *C. langsdorffii *against a representative panel of microorganisms responsible for periodontitis. Copalic acid was the most active diterpene, displaying a very promising MIC value (3.1 μg mL^−1^) against the key pathogen (*Porphyromonas gingivalis*) involved in this infectious disease. Moreover, it did not exhibit cytotoxicity when tested in human fibroblasts. In another paper, Souza *et al*. [126] reported that copalic acid was active against the main microorganisms responsible for dental caries: *Streptococcus salivarius*, *S. sobrinus*, *S. mutans*, *S. mitis*, *S. sanguinis *and *Lactobacillus casei*. 

Hardwickiic acid is another diterpene that is very common in copaiba oleoresins, being detected in about 42% of them [127]. Some studies have been performed with this diterpene to determine its antibicrobial activity [128]. It has shown significant qualitative antibacterial activity against *B. subtilis*, *S. aureus* and *Mycobacterium smegmatis*. Moreover, hardwickiic acid, isolated from the stem bark of *Irvingia gabonensis*, inhibited the growth of several bacteria and fungus species using dilution methods [129]. However, in a recent study, when it was assayed against a collection of Gram-negative multidrug-resistant bacteria, the diterpene was inactive [130].

Kaurenoic acid (*ent*-kaur-16-en-19-oic acid) was first described in 1971 by Ferrari *et al*. [131]. However, this acid was only detected in copaiba oleoresins in 1998 by Braga *et al.* [132] who isolated it from *C. cearensis* using ion-exchange chromatography. Although this diterpene is present in about 30% of copaiba oleoresins [124], it sometimes cannot be detected by GC because its retention time is similar to with that of copalic acid, resulting in co-elution. Therefore, it was not possible to distinguish the kaurenoic (or the copalic) acid [133]. Several pharmacological studies were performed with kauran acid, to determine it uterine muscle relaxant [134], anti-inflammatory [135], bactericidal [86], and cytotoxicity [136] effects, activity against *Trypanosoma cruzi *tripomastigotes [137], genotoxicity induction [138], and vasodilatory effects [139].

The uterine relaxant effects of kaurenoic acid were reported by Cunha *et al*. [134], who isolated this diterpene from *C. langsdorffii* oleoresin. Accoding to these authors, kaurenoic acid exerts this relaxant effect principally through calcium blockade and in part, by the opening of ATP-sensitive potassium channels. Another study investigated the mechanisms involved in the vasorelaxant action of kaurenoic acid in isolated aortic rings in rats [139].

According to Cavalcanti *et al*. [138], kaurenoic acid has DNA damaging activity in cultured Chinese hamster fibroblasts (V79 cells) under the conditions of the Comet assays. Costa-Lotufo *et al.* [136] indicated the potential cytotoxicity of kaurenoic acid by the destruction of sea urchin embryos, the inhibition of tumor cell growth and the hemolysis of mouse and human erythrocytes.

Anti-inflammatory activity was reported by Paiva *et al*. [135] who isolated the kaurenoic acid from oleoresin of *C. langsdorffii* Desf. In this study, it was observed that kaurenoic acid prevented tissue damage in the rat model of acetic acid colitis, an effect which the authors verified through macroscopic, histological and biochemical changes.

It has been reported also that kaurenoic acid, isolated from *C. paupera* oleoresin, showed antibacterial activity against *B. subtilis*, *S. aureus*, *S. epidermidis *[86]. In other study, kaurenoic acid, and some of its derivatives, showed *in vitro *activity against trypomastigote forms of *Trypanosoma cruzi *[137], with kaurenoic acid derivatives presenting less side effects than the acid. 

Souza *et al. *[140] showed that kaurenoic and polyalthic acids (the latter less active) were capable of promoting inhibition of rhodamine 6G efflux in *Saccharomyces cerevisiae *with Pdr5p enzyme (protein that confers multiple drug resistance).

Another diterpene detected in copaiba oleoresin, 3α-hydroxy-kaurenoic acid presented higher fungitoxic activity against *Botrytis cinerea *(a phytopathogenic fungus that attacks the flowers, fruits, leaves, and stems of several plants). The authors suggest that the diterpene probably acts by inhibiting germination and mycelium growth this fungus [141].

Larvicidal activity has also been reported against *A. aegypti *larvae, of two labdane diterpenes isolated from *C. reticulata *oleoresin: (-)-3β-hydroxilabd-8(17)-13-dien-15-oic acid and 3-β-acetoxy-labd-8(17)-13-dien-15-oic acid [142]. In another study, this latter diterpene was reported to cause death of the *A. aegypti *larvae by cell destruction in the midgut [143]. Also, another diterpene present in copaiba oleoresin, kovalenol, showed potent antitumor activity against IMC carcinoma (murine tumor) in mice [144].

## 4. Conclusions

Although many papers have been published on the chemical composition of copaiba oleoresins, several questions remain unsolved, such as the fingerprint of the chemical composition of the different species and the presence of biomarkers, probably a combination of sesquiterpenes and diterpernic acids. Ethnopharmacological studies indicate many activities that are still not fully understood through pharmacological experiments. Also, the activities of the isolated compounds do not explain the strong activities of crude oleoresins. Indeed, several substances have being described, and new biological studies have been published that go some way to unraveling the action mechanism of the isolated sesquiterpenes and diterpenes. All these topics still require further investigation, as copaiba oil is a resource on which there is still much work to be done.
